# 

**Published:** 1851-07

**Authors:** 


					﻿ARTICLE V.
CHOLERA.
The cholera appears to be prevailing to a limited extent in
the region of St. Louis. In that city there have been about fif-
teen deaths from it reported daily for two weeks. We learn
that it has been prevailing at Alton, Quincy, Springfield and
some other points in the southern part of Illinois.
As yet it has not made its appearance in Chicago ; some
cases, probably of cholera morbus, have excited the fears of
the nervous. An agent has been stationed at LaSalle, by the
Board of Health, to give notice of the first approach of the
disease from below, when, the Mayor informs us, a quarantine
will at once be established. If well conducted we shall have
strong hope that we may be spared a visitation of the
pestilence the present season.
				

